# Progression of Fatigue in Early Parkinson’s Disease: A 3-Year Prospective Cohort Study

**DOI:** 10.3389/fnagi.2021.701906

**Published:** 2021-10-20

**Authors:** Ruwei Ou, Yanbing Hou, Kuncheng Liu, Junyu Lin, Zheng Jiang, Qianqian Wei, Lingyu Zhang, Bei Cao, Bi Zhao, Wei Song, Huifang Shang

**Affiliations:** Laboratory of Neurodegenerative Disorders, Department of Neurology, National Clinical Research Center for Geriatrics, West China Hospital, Sichuan University, Chengdu, China

**Keywords:** Parkinson’s disease, fatigue, anxiety, sleep disturbance, apathy

## Abstract

**Objective:** To explore the frequency, evolution, associated factors, and risk factors of fatigue over 3-year of prospective follow-up in a cohort of patients with early Parkinson’s disease (PD).

**Methods:** A total of 174 PD patients in the early stage were enrolled and quantitively assessed motor and non-motor symptoms using comprehensive scales including the Fatigue Severity Scale (FSS) annually. Each subject was categorized as PD with and without fatigue based on a cut-off mean value of 4 using FSS. The generalized estimating equation (GEE) was utilized to investigate the associated factors, and the stepwise binary logistic regression model was performed to explore the predictors.

**Results:** The frequency of fatigue was slightly changed (ranging from 35.1 to 40.4%) during the 3-year follow-up. The changed pattern of the frequency of fatigue was similar to that of anxiety. Fatigue was significantly associated with nocturnal sleep disorders (B 2.446, *P* < 0.001), high Hamilton Anxiety Rating Scale (HAMA) score (B 1.072, *P* = 0.011), and high Unified PD Rating Scale (UPDRS) III score (B 1.029, *P* = 0.003) over time. High UPDRS III score [odds ratio (OR) 1.051, *P* = 0.015] at baseline increased the risk of developing fatigue after 1-year; high LEDD (OR 1.002, *P* = 0.037) increased the risk of developing fatigue after 2-year; and high LEDD (OR 1.003, *P* = 0.049) and high HAMA score (OR 1.077, *P* = 0.042) increased the risk of developing fatigue after 3-year.

**Conclusion:** Our present study provided evidence of the longitudinal evolution of fatigue in patients with early PD and help clinical management of fatigue.

## Introduction

Fatigue is a common and poorly understood non-motor symptom in patients with Parkinson’s disease (PD), occurring in all stages of the disease even in the pre-motor period, and its prevalence often increases with the disease progression (Kluger et al., 2016). Although the universally accepted definition of fatigue in PD has not been achieved, fatigue is usually considered as a sense of tiredness, lack of energy, or total body give out (Krupp et al., 1989). More than half of PD patients reported that fatigue occurring after the onset of PD was qualitatively different from that experiencing before the onset of PD (Friedman et al., 2007). Fatigue is one of the most disabling symptoms in PD, limiting the ability to maintain hobbies and participate in social activities (Kluger et al., 2016), and further impact the quality of life (Havlikova et al., 2008).

Previously conducted studies on fatigue in PD are mainly cross-sectional and case-control designs. In the retrospective analysis, the estimated prevalence of fatigue varied from 33 to 58% (Siciliano et al., 2018). It was reported to be associated with older age (Skorvanek et al., 2015), female sex (Kummer et al., 2011; Stocchi et al., 2014), motor disability (Alves et al., 2004; Schifitto et al., 2008; Stocchi et al., 2014), cognitive impairment (Pereira et al., 2016), neuropsychiatric symptoms (Herlofson et al., 2012; Stocchi et al., 2014; Siciliano et al., 2017), and sleep disorders (Okuma et al., 2009; Stocchi et al., 2014), but with great heterogeneity. Among them, female sex, depression, and cognitive impairment were also identified to be associated with fatigue in the longitudinal analysis (Ongre et al., 2020), however, the improvement of fatigue from baseline to 1-year later were found not to be associated with the changes in disease severity, depressive symptoms, sleep problems, apathy, and cognitive impairment (Ongre et al., 2017). Some evidence indicates that fatigue seems like an independent symptom of PD with no relation to other motor or non-motor symptoms (Herlofson and Larsen, 2002; Alves et al., 2004; Okuma et al., 2009). Moreover, the pathophysiology of fatigue in PD is still unclear and the evidence for the management of fatigue using pharmacologic or non-pharmacologic treatments is insufficient (Franssen et al., 2014).

Evidence from the longitudinal study design is important and useful for early intervention and clinical management. To date, however, such prospective studies (Ongre et al., 2017, 2020) on fatigue in PD with repeated measurements are scarce. There is a clear need for acquiring more information about the dynamic evolution and predictors of fatigue in the early stage of PD. Therefore, in the current prospective study, we aimed to recruit and follow a cohort of early PD patients over 3 years to determine the prevalence, evolution, associated factors, and predictors for risk of fatigue in early PD.

## Patients and Methods

### Study Design and Population

The study was approved by the Ethics Committee of Sichuan University West China Hospital and written informed consent was obtained from all the participants. The current study is a part of the ongoing prospective cohort study on the clinical progression and prognosis of Chinese PD patients who performed at Department of Neurology, Sichuan University. This protocol started in February 2014 and focused on recruiting PD patients with a disease duration of < 3 years. PD was diagnosed according to the United Kingdom PD Society Brain Bank Clinical Diagnostic Criteria (Hughes et al., 1992) and the Movement Disorder Society clinical diagnostic criteria for PD (Postuma et al., 2015). The exclusion criteria were listed as follows: (1) patients with cognitive impairment, as assessed by the Montreal Cognitive Assessment (MOCA) score < 22 (Yu et al., 2012); (2) patients with motor complications; and (3) patients with Hoehn and Yahr (H&Y) stage ≥ 3.

Standardized examinations and repeated assessments of patients were executed each year by trained neurologists in our movement disorder center. Three months before and 6 months after the next visit are allowed. In the prospective cohort study, the analysis was performed based on the assessments at baseline, 1-, 2-, and 3-year follow-up. Of the 302 patients we included initially, 128 were excluded due to lack of fatigue assessment at baseline (*n* = 117) or insufficient data at the follow-up visit (*n* = 11), leaving 174 patients eligible for the data analysis. All the PD patients had confirmed the diagnosis of PD during follow-up. The sample size and power were enough based on a previous report (Liu and Liang, 1997).

### Clinical Assessments

At baseline, the following demographic and clinical data were collected: sex, age, disease duration, and schooling years. The antiparkinsonian medication regimen was recorded at each visit, which was converted to the total levodopa equivalent daily doses (LEDD) based on a previous review (Tomlinson et al., 2010).

A detailed series of neurological examinations at baseline and during follow-up were conducted. The motor symptom was evaluated by the Unified PD Rating Scale (UPDRS) part III (Movement Disorder Society Task Force on Rating Scales for Parkinson’s, Disease, 2003) and the H&Y stage (Hoehn and Yahr, 1967). The executive function was evaluated using the Frontal Assessment Battery (FAB) (Dubois et al., 2000), while the global cognitive function was evaluated using the MOCA (Nasreddine et al., 2005). The prevalence of Rapid eye movement (REM) behavior disorder (RBD) was calculated from the percentage of patients who obtained a score of ≥ 5 in the RBD Screening Questionnaire (RBDSQ) (Stiasny-Kolster et al., 2007). The 24-item version of the Hamilton Depression Rating Scale (HAMD) (Moberg et al., 2001) was used to evaluate depressive symptoms, and the Hamilton Anxiety Rating Scale (HAMA) (Hamilton, 1959) was used to evaluate anxious symptoms. Apathy was evaluated by the Lille Apathy Rating Scale (LARS) (Leentjens et al., 2008). Based on the above assessments, patients were classified into the absence of depression (HAMD score ≤ 20) and presence of depression (HAMD score > 20) groups (Gibbons et al., 2012), absence of anxiety (HAMA score ≤ 14) and presence of anxiety (HAMA score > 14) groups (Kummer et al., 2010), or absence of apathy (LARS < −21) and presence of apathy (LARS ≥ −21) groups (Sockeel et al., 2006). The presence of nocturnal sleep disorders (including fragmented sleep and insomnia) was based on the self-reported by each participant. Specific scales, such as PD Sleep Scale 2nd version, were not used to assess sleep disorders because they had not been designed to be applied at baseline.

### Definition of Fatigue

Fatigue was annually evaluated by the Fatigue Severity Scale (FSS) (Krupp et al., 1989), which is consists of 9 items, yielding a score range between 1 and 7. Higher scores indicate a higher level of fatigue. The mean score of FSS was calculated. The prevalence of fatigue was calculated from the percentage of patients who obtained a mean score > 4 in the FSS (Leentjens et al., 2008).

### Statistical Analyses

Statistical analyses were performed in the Statistical Package for the Social Sciences (SPSS) version 22.0. All statistical tests were two-tailed, and *P*-values < 0.05 were considered statistically significant. The continuous variables were presented as means and standard deviation (SD) if normally distributed and as median and interquartile range (IQR) if non-normally distributed. The categorical variables were reported as counts (percentages).

The generalized estimating equation (GEE) was applied to explore the associated factors of fatigue. The GEE model was based on all patients in the cohort and included all consecutive examinations during follow-up to allow for correlation between repeated measurements of the same patients. An exchangeable working correlation structure was selected based on the quasi-likelihood under the independence model criterion. The presence or absence of fatigue was set as the dependent variable in the model. The following parameters including age, sex, disease duration, education, LEDD, use of levodopa, use of dopamine agonist, use of monoamine oxidase type B (MAO-B) inhibitor, use of antidepressant, sleep disorders, RBD, UPDRS III score, FAB score, MOCA score, HAMD score, HAMA score, LARS score, and follow-up time in years were included as the independent variables. The procedures of analysis were first performed including only one covariate at a time (unadjusted model) and then included covariates with *P-*values < 0.1 or those that were possibly associated with fatigue (adjusted model).

The binary logistic regression models were used to investigate the clinical predictors for fatigue in PD. The analysis was based on the patients who had no fatigue at baseline. The clinical outcome was the new occurrence of fatigue during follow-up. In the multivariate models, based on the results of the GEE analysis and experience in clinical practice, age, sex, disease duration, LEDD, MOCA score, HAMD score, HAMA score, LARS score, sleep disorders, and UPDRS III score were included as covariates. Each dependent variable’s variance inflation factor (VIF) was calculated to diagnose the multicollinearity, with a value > 5 suggested the presence of multicollinearity.

### Data Availability

Anonymized data can be obtained by request from qualified investigators for purposes of replicating procedures and results.

## Results

### Baseline Characteristics

The demographic and baseline clinical features of enrolled 174 PD patients (51.1% males) are listed in Table 1. The mean age of enrolled patients at baseline was 57.7 (SD 10.8) years, with a mean PD duration of 1.5 (SD 0.8) years. At baseline, eighty-three patients (47.7%) received antiparkinsonian therapy, and the mean LEDD was 144.8 (SD 185.0) mg/day. After 3 years, all patients were treated with antiparkinsonian drugs, and the mean LEDD was 504.5 (SD 229.6) mg/day.

**TABLE 1:** Demographic and clinical features of PD patients.

	**Baseline**	**1-year**	**2-year**	**3-year**
Number of samples	174	174	172	134
Age, years, mean (SD), [95% CI]	57.7 (10.8), [56.1–59.3]	59.0 (10.8), [57.4–60.6]	60.3 (10.8), [58.7–62.0]	61.4 (11.2), [59.4–63.3]
Disease duration, median (IQR), [95% CI]	1.5 (1.3), [1.4–1.7]	2.7 (1.6), [2.7–3.0]	4.0 (1.5), [3.9–4.3]	5.0 (1.6), [5.0–5.4]
Male sex, n (%), [95% CI]	89 (51.1), [1.4–1.6]	89 (51.1), [1.4–1.6]	87 (50.6), [1.4–1.6]	70 (52.2), [1.4–1.6]
Education, median (IQR), [95% CI]	12 (6), [10.5–11.7]	12 (6), [10.5–11.7]	12 (6), [10.5–11.7]	12 (6), [10.3–11.7]
LEDD, mg/day, median (IQR), [95% CI]	0 (300), [117.1–172.5]	300 (300), [305.5–357.0]	450 (250), [400.9–460.9]	500 (275), [465.3–543.7]
Use of levodopa, n (%), [95% CI]	64 (36.8), [0.3–0.4]	118 (67.8), [0.6–0.8]	137 (79.7), [0.7–0.9]	115 (85.8), [0.8–0.9]
Use of dopamine agonist, n (%), [95% CI]	45 (25.9), [0.2–0.3]	116 (66.7), [0.6–0.7]	144 (83.7), [0.8–0.9]	116 (86.6), [0.8–0.9]
Use of MAO-B inhibitor, n (%), [95% CI]	11 (6.3), [0–0.1]	8 (4.6), [0–0.1]	20 (11.6), [0.1–0.2]	20 (14.9), [0.1–0.2]
Use of anti-depressant, n (%), [95% CI]	6 (3.4), [0–0.1]	5 (2.9), [0–0.1]	14 (8.1), [0–0.1]	14 (10.4), [0–0.1]
FAB score, median (IQR), [95% CI]	17 (2), [16.4–16.9]	17 (2), [16.3–16.9]	17 (2), [16.3–16.8]	17 (2), [16.2–16.8]
MOCA score, median (IQR), [95% CI]	27 (4), [26.0–26.7]	27 (4), [26.1–27.0]	27 (4), [26.0–26.8]	27 (4), [25.8–26.8]
HAMD score, median (IQR), [95% CI]	6 (10), [6.7–8.8]	6 (8), [6.2–8.0]	6 (8), [6.2–8.1]	7 (7), [6.6–8.7]
HAMA score, median (IQR), [95% CI]	4 (8), [4.9–6.6]	5 (7), [5.3–7.1]	5 (7), [5.0–6.6]	6 (7), [5.6–7.5]
LARS score, median (IQR), [95% CI]	−30 (10), [−28.7-−25.7]	−29 (9), [−28.5-−26.3]	−29 (11), [−27.8-−25.2]	−28 (11), [−27.0-−24.1]
FSS score, median (IQR), [95% CI]	28 (32), [36.4–31.6]	31 (30), [28.5–33.6]	29 (31), [27.2–32.4]	27 (32), [26.4–32.0]
UPDRS III score, median (IQR), [95% CI]	22 (14), [21.7–24.9]	24 (15), [23.7–26.8]	26 (14), [25.9–29.1]	27 (12), [26.8–30.8]
H&Y, median (IQR), [95% CI]	2 (0), [1.8–2.0]	2 (0), [2.0–2.1]	2 (0), [2.1–2.2]	2 (0.5), [2.1–2.3]

*PD, Parkinson’s disease; LEDD, levodopa equivalent daily dose; MAO-B, Monoamine oxidase type B; FAB, Frontal Assessment Battery; MOCA, Montreal Cognitive Assessment; HAMD, Hamilton Depression Rating Scale; HAMA, Hamilton Anxiety Rating Scale; LARS, Lille Apathy Rating Scale; FSS, Fatigue Severity Scale; UPDRS, Unified Parkinson’s Disease Rating Scale.*

### Frequency and Evolution of Apathy

**FIGURE 1:**
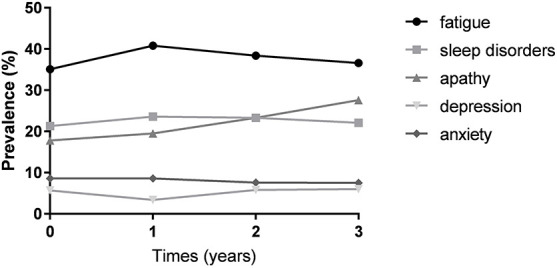
The prevalence of fatigue in patients with PD at each visit. The prevalence of fatigue in patients with PD ranged from 35.1 to 40.8% in the early stage within 3 years. The changed pattern of the prevalence of fatigue was similar to that of anxiety rather than that of sleep disorders, depression, and apathy. PD, Parkinson’s disease.

**FIGURE 2:**
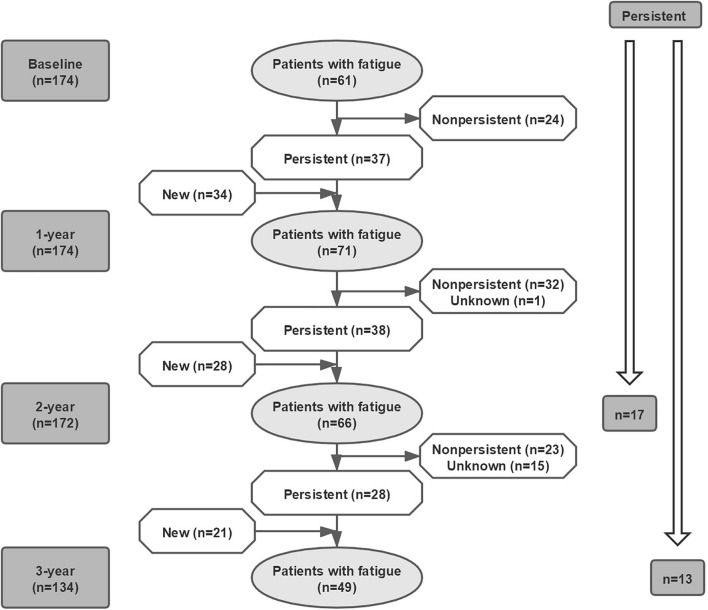
Evolution of fatigue in patients with PD over time. The presence of fatigue was not persistent over 3-year. The number of persistent fatigue from baseline to 1-, 2-, and 3-year were 37, 17, and 13, respectively. PD, Parkinson’s disease.

Figure 1 showed that the observed frequency of fatigue in patients with PD over 3-year. Of 174 patients, 61 reported fatigue at baseline (35.1%). During follow-up, the frequency of fatigue increased to 71/174 (40.8%) after 1-year, 66/172 (38.4%) after 2-year, and 49/134 (36.6%) after 3-year. The changed pattern of the prevalence of fatigue was similar to that of anxiety; whereas, it was different from that of sleep disorders, depression, and apathy (Figure 1). Fatigue was not always persistent from one visit to the next in every patient during the 3-year study period (Figure 2). The number of persistent fatigue from baseline to 1-, 2-, and 3-year were 37, 17, and 13, respectively.

### Associated Factors of Fatigue in Parkinson’s Disease Over Time

The associated factors for fatigue in PD over time are presented in Table 2. The GEE analyses indicated that fatigue was independently associated with nocturnal sleep disorders [B 2.446, 95% confidence interval (CI) 1.580–3.788, *P* < 0.001], high HAMA score (B 1.072, 95%CI 1.016–1.131, *P* = 0.011), and high UPDRS III score (B 1.029, 95%CI 1.010–1.049, *P* = 0.003) over time (adjusted model). In the GEE model, the goodness of fit was 786.823.

**TABLE 2:** Factors associated with fatigue in patients with PD.

	**Unadjusted model**			**Adjusted model**		
	**B**	**95%CI**	***P*-value**	**B**	**95%CI**	***P*-value**
Age	0.990	0.970–1.010	0.320	0.981	0.960–1.002	0.078
Male sex	0.819	0.541–1.239	0.344	0.783	0.520–1.180	0.164
Disease duration	1.023	0.927–1.128	0.656	0.906	0.794–1.035	0.145
Education	0.974	0.922–1.030	0.354			
LEDD	1.001	1.000–1.001	0.031*	1.001	1.000–1.002	0.153
Use of levodopa	1.145	0.826–1.587	0.416			
Use of dopamine agonist	1.140	0.848–1.532	0.386			
Use of MAO-B inhibitor	0.766	0.441–1.330	0.343			
Use of antidepressant	0.979	0.438–2.191	0.960			
FAB	0.917	0.833–1.009	0.076	0.957	0.837–1.093	0.515
MOCA	0.948	0.892–1.007	0.083	0.990	0.916–1.070	0.806
Sleep disorders	3.450	2.319–5.133	<0.001*	2.446	1.580–3.788	<0.001*
RBD	0.984	0.647–1.496	0.940			
HAMD	1.084	1.052–1.117	<0.001*	1.001	0.951–1.049	0.969
HAMA	1.109	1.072–1.148	<0.001*	1.072	1.016–1.131	0.011*
LARS	1.024	1.006–1.043	0.009*	1.010	0.987–1.033	0.391
UPDRS III	1.038	1.022–1.053	<0.001*	1.029	1.010–1.049	0.003*
Follow-up time in years	1.017	0.891–1.162	0.798			

PD, Parkinson’s disease; LEDD, levodopa equivalent daily dose; MAO-B, Monoamine oxidase type B; FAB, Frontal Assessment Battery; MOCA, Montreal Cognitive Assessment; RBD, Rapid eye movement behavior disorder; HAMD, Hamilton Depression Rating Scale; HAMA, Hamilton Anxiety Rating Scale; LARS, Lille Apathy Rating Scale; UPDRS, Unified Parkinson’s Disease Rating Scale. *Significant difference.

### Predictors of Fatigue in Parkinson’s Disease

The predictors for fatigue in PD are presented in Table 3. In the three multivariate models, all the VIF for each variable was less than 5. High UPDRS III score [odds ratio (OR) 1.051, 95%CI 1.010–1.094, *P* = 0.015] increased the risk of developing fatigue after 1-year; high LEDD (OR 1.002, 95%CI 1.000–1.005, *P* = 0.037) increased the risk of developing fatigue after 2-year; and high LEDD (OR 1.003, 95%CI 1.000–1.005, *P* = 0.049) and high HAMA score (OR 1.077, 95%CI 1.003–1.157, *P* = 0.042) increased the risk of developing fatigue after 3-year.

**TABLE 3:** Predicted factors for the development of fatigue in patients with PD.

		**Occurrence of fatigue**		
		**OR**	**95%CI**	***P*-value**
From baseline to 1-year (*n* = 113)	UPDRS III	1.051	1.010–1.094	0.015*
From baseline to 2-year (*n* = 111)	LEDD	1.002	1.000–1.005	0.037*
From baseline to 3-year (*n* = 84)	LEDD	1.003	1.000–1.005	0.049*
	HAMA	1.077	1.003–1.157	0.042*

PD, Parkinson’s disease; UPDRS, Unified Parkinson’s Disease Rating Scale; LEDD, levodopa equivalent daily dose; HAMA, Hamilton Anxiety Rating Scale. *Significant difference.

## Discussion

The strength of the present study is that we included a large sample of patients with early PD to explore the frequency, evolution, associated factors, and predicted factors of fatigue using a validated scale to definite the symptom. In the prospective cohort study, we found that fatigue was frequent and non-persistent in patients with early PD, which was linked to motor severity, sleep disorders, and anxiety symptom. We also found that severer disability and severer anxiety symptoms at an early stage are predictors for the development of fatigue in PD. Our present study provided evidence of the longitudinal evolution of fatigue in Chinese patients with early PD and might have implications for early intervention and clinical management. However, the generalizability of our results needs to be further validated by larger Chinese or Western cohorts.

The frequency of fatigue varied from 33 to 58% in PD patients based on previous cross-sectional and case-control studies (Siciliano et al., 2018), probably reflecting the differences in the study design, especially the definition of fatigue, as well as in the stages and durations that patients were enrolled. In the present study, we found that the frequency of fatigue was common (range between 35 and 41%) in the early stage of PD, which supports that fatigue is one of several common non-motor symptoms that is experienced by PD patients in the early stage.

Fatigue was reported to respond to dopaminergic drugs such as levodopa (Lou et al., 2003), suggesting that dopaminergic deficits are involved in the development of fatigue in PD. In the current study, more severe motor disabilities or higher LEDD predicting fatigue support the above finding. In addition, the involvement of dopamine in fatigue is verified by a previous study that reported that fatigue in patients with PD is associated with an increased α-synuclein oligomer level in the cerebrospinal fluid (Zuo et al., 2016). However, only partially response to dopaminergic treatment and lack of association with [^123^I]-β-CIT striatal measurements suggest that the dopaminergic pathway is one of the multi-mechanisms contributing to the pathogenesis of fatigue in PD (Schifitto et al., 2008). The non-persistent property of fatigue observed in the current study also suggests that dopaminergic mechanisms only partially contribute to the development of fatigue in PD.

Although most cross-sectional studies did not find a relationship between fatigue and anxiety in PD, our finding that the association between the high level of anxiety symptoms and the development of fatigue was supported by two previous cross-sectional studies (Cochrane et al., 2015; Siciliano et al., 2017) using either Parkinson’s Anxiety Scale or self-reported assessment to measure anxiety. More importantly, in the current study, we found that a similar fluctuated pattern of the prevalence of fatigue with that of anxiety over time, which suggests that the relationship between apathy and anxiety is very close. Furthermore, we also addressed the causal relationship between fatigue and anxiety. In the current study, anxiety might contribute to the subjective perception of fatigue, demonstrating that both the non-motor symptoms share similar underlying mechanisms. The relationship between fatigue and anxiety in PD could be explained by the fact that both symptoms might derive from a dysfunction of the dopaminergic mesocorticolimbic system, a region that seems to play a role in the control of mood and motivation (Marin, 1991). In addition, a Positron Emission Tomography study found that fatigue in PD is associated with the reduced serotonergic function of the basal ganglia and limbic structures (Pavese et al., 2010), also supporting a common neural pathway between fatigue and anxiety in PD.

However, our cohort study did not find a relationship between fatigue and depressive symptom, though some cross-sectional studies found that depression was associated with fatigue in PD (Schifitto et al., 2008; Stocchi et al., 2014). Fatigue has been identified to be independent of depression on the basis that all the non-motor symptoms can be clearly distinguished in PD, such that patients with “pure depression” or with “pure fatigue” can be found (Skorvanek et al., 2015). In addition, our longitudinal study is inconsistent with the view that female sex was associated with fatigue in PD (Barone et al., 2009; Martinez-Martin et al., 2012), which also supports the fact that depression is not associated with fatigue in PD because female PD patients affected by depression were found to have a higher rate of fatigue (Watanabe et al., 2008). Due to the complicated relationship between fatigue, anxiety, and depression, further pathologic studies will help to clarify this issue.

Although sleep disorders and fatigue are reported as independent non-motor symptoms in PD (Friedman et al., 2007), we found that the two symptoms were closely associated with each other in our cohort, which was supported by two previous cross-sectional studies (Okuma et al., 2009; Stocchi et al., 2014) that reported that sleep disorders and fatigue showed as “symptom cluster” in PD. Patients with sleep disorders often feel tired in the morning, so it is reasonable to report fatigue during the daytime. However, some patients who did not have sleep disorders also reported fatigue at each visit, indicating that the pathogenesis of fatigue in PD is multifactorial.

Some limitations should be acknowledged. First, we did not include a group of healthy controls to compare the evolution of fatigue in patients with PD. Second, all the participants were recruited through a tertiary referral center in West China, and the results should be further verified by a multi-center study. Third, nearly half of the patients at baseline were receiving drug treatment, leading to unable to analyze the progression of “pure fatigue” in PD. Forth, a relatively short observation of disease progression of patients is not sufficient to explore the long-term evolution of fatigue in PD. Fifth, nocturnal sleep disorders were self-reported by the participants. Objective measures on nocturnal sleep disorders such as polysomnography (PSG) should be applied in future studies. Sixth, the evaluation of motor severity is based on the UPDRS rather than the MDS version of UPDRS.

## Conclusion

Our study reported the onset and development of fatigue in the early patients with PD, which provided evidence of the longitudinal evolution of fatigue in early PD and had implications for the management of fatigue.

## Data Availability Statement

The raw data supporting the conclusions of this article will be made available by the authors, without undue reservation.

## Ethics Statement

The studies involving human participants were reviewed and approved by the Ethics Committee of Sichuan University West China Hospital. Written informed consent to participate in this study was provided by the participants’ legal guardian/next of kin.

## Author Contributions

RO: research project conception, organization, execution, statistical analysis and design, and writing of the manuscript draft. RO, YH, KL, JL, ZJ, QW, LZ, BC, BZ, and WS: patients’ enrollment and follow up. HS: research project conception, statistical analysis, review and critique, and manuscript revision of the draft. All authors contributed to the article and approved the submitted version.

## Conflict of Interest

The authors declare that the research was conducted in the absence of any commercial or financial relationships that could be construed as a potential conflict of interest.

## Publisher’s Note

All claims expressed in this article are solely those of the authors and do not necessarily represent those of their affiliated organizations, or those of the publisher, the editors and the reviewers. Any product that may be evaluated in this article, or claim that may be made by its manufacturer, is not guaranteed or endorsed by the publisher.
